# Familial genetic and environmental transmission of depression: A multi‐informant twin family study

**DOI:** 10.1002/pchj.751

**Published:** 2024-04-15

**Authors:** Qingwen Ding, Yueyue Zhou, Shuting Yu, Xiaobing Cui, Xiaoyu Wang, Xinying Li

**Affiliations:** ^1^ CAS Key Laboratory of Mental Health, Institute of Psychology Chinese Academy of Sciences Beijing China; ^2^ Department of Psychology University of Chinese Academy of Sciences Beijing China; ^3^ Department of Psychology Henan University Kaifeng China

**Keywords:** adolescents, depression, familial transmission, genetics, twins

## Abstract

The phenomenon of familial clustering in depression is well established, yet the mechanisms by which depression is transmitted within families remain poorly understood. In the current study, we investigate the familial genetic and environmental transmission of depression by incorporating data from both adolescent twins and their parents. A total of 987 twin families were recruited from the Beijing Twin Study. Depression assessments were conducted for both adolescents and their parents. Twins' depression was assessed through reports from both the twins themselves and their parents, while parental depression was assessed by parental self‐report. We employed a nuclear twin family model to examine genetic and environmental influences on adolescent depression. Our results, based on both self‐ and parent‐report, demonstrate significant additive and dominant genetic influences on depression. We also found mild yet significant sibling environmental influences, while familial environmental influences were absent. Notably, parent‐reported depression showed higher heritability but lower unique environmental influences compared with self‐reported depression. These results highlight the important role of genetic transmission and sibling environmental transmission in explaining depression. Our study delineates the underlying mechanism of familial transmission in depression and can inform early treatments to halt transmission during adolescence.

## INTRODUCTION

Familial clustering in depression has been extensively documented in previous research, indicating substantial transmission among family members (Buist et al., 2019; Harold et al., 2011; Heun & Hein, 2007; Merikangas et al., 2014; Middeldorp et al., 2005; Tully et al., 2008). Depression can be transmitted between parents and children, as well as between siblings, with both genetic and environmental factors intricately involved in this process. However, how and the extent to which depression is genetically and environmentally transmitted within families remain poorly understood. Depressive symptoms emerge early and peak in adolescence, affecting up to 34% of adolescents worldwide (Shorey et al., 2022). Adolescent depressive symptoms are associated with long‐term poor mental and psychosocial outcomes (Clayborne et al., 2019). The examination of familial transmission when children are in adolescence is of particular significance, as it can provide valuable insights into the etiology of depression and inform early treatments to mitigate the transmission.

The classical twin (CT) model is a commonly employed approach to explore the genetic and environmental influences contributing to adolescent depression. Specifically, it utilizes data from dizygotic (DZ) and monozygotic (MZ) twins to detect three types of sources, namely additive genetic (A), common environmental (C), and unique environmental (E) influences. In this model, A represents the genetic relatedness between twins, C represents the environmental relatedness, and E represents the environmental difference. Although CT studies have yielded abundant results (Chen et al., 2014; Eley & Stevenson, 1999; Rice et al., 2002; Scourfield et al., 2003; Silberg et al., 2001; Zheng et al., 2016), estimates of these genetic and environmental contributions vary widely: A has been estimated to be from 0% to 80%, C from 0% to 57%, and E from 20% to 79%. The discrepancies in these estimates can be attributed to the limitations inherent in the CT model. Because of the absence of parental depression data in this model, several important factors contributing to familial transmission have been overlooked.

For instance, the CT research commonly uses A to represent genetic transmission, as it is shared by all family members. It is possible, however, that both additive alleles (i.e., additive genetic influences) and interactions between alleles (i.e., dominant genetic influences, D) inherited from parents contribute to depression. Although D does not create similarity between parents and children, it plays a significant role in contributing to sibling similarity (Keller et al., 2010). Unfortunately, without parental data, the CT model is not able to estimate D and C together, leading to the omission of D. Ignoring D can lead to an overestimation of A (Keller & Medland, 2008). Additionally, genetic transmission can be influenced by assortative mating between parents, whereby individuals tend to mate with those who have a similar phenotype (Luo, 2017). This can lead to a stronger correlation between the phenotypes of fathers and mothers. Previous studies have indeed found significant couple similarities in terms of depression (Desai et al., 2012; Torvik et al., 2022). The presence of assortative mating can result in a decrease of A estimates in the CT model (Keller et al., 2010).

Estimates of environmental transmission in the CT model are also rough and indirect. The CT model uses C to indicate the environmental transmission between twins. It fails to adequately distinguish between parent–child environmental transmission, which is shared by all family members, and sibling environmental transmission, which is shared only by siblings. In fact, it is possible that both parent–child and sibling socialization contribute to depression. Previous studies have shown that several familial environmental factors (e.g., family socioeconomic status and family relationships) and sibling environmental factors (e.g., sibling interaction, peer relationships, and school climate) play a significant role in influencing adolescent depression (Buist et al., 2019; Jia et al., 2009; Mendelson et al., 2010; Pino et al., 2018; Queen et al., 2013; Yang et al., 2020). However, the extent to which depression is environmentally transmitted from parents to children and between siblings has not been examined. A comprehensive examination of the relative proportions can help in prioritizing specific targets in clinical practice.

Moreover, the correlation between genetic and environmental influences, such as passive gene–environment correlation, makes it more difficult to understand depression transmission (Warrier et al., 2021). Passive gene–environment correlation conceptualizes the association between children's inherited genes and the rearing environment (Knafo & Jaffee, 2013). This correlation occurs when parents pass on depressive genes to their offspring and, at the same time, provide a home environment that impacts their offsprings’ depression. Within the CT model, passive gene–environment correlation can result in an increase of C estimates and a decrease of A and D estimates (Keller et al., 2010).

Finally, different informant sources, such as self‐reported depression and parent‐reported depression, can lead to discrepant evaluations on familial transmission. In CT research, parent‐reported depression usually exhibits stronger A but lower E compared with self‐reported depression (Chen et al., 2014; Rice et al., 2002; Scourfield et al., 2003). When exploring how depression transmits within family members, the inclusion of multi‐informant data can improve sensitivity to rater discrepancies (De Los Reyes, 2011).

To address these limitations, this study used a nuclear twin family (NTF) model (Keller et al., 2009) to investigate the familial genetic and environmental transmission of depression. In addition to depression data for twins, the NTF model also gathers depression data for their parents. This more comprehensive data resource allows for more differentiated and precise estimates of genetic and environmental influences (Keller et al., 2010). For genetic transmission, the NTF model considers both A and D. For environmental transmission, it divides C into familial environmental influences transmitted from parents to all children and sibling environmental influences transmitted only between siblings. Furthermore, assortative mating and passive gene–environment correlation are also considered in this model in order to obtain reliable results. Finally, we utilized multi‐informant data, including reports of adolescents' depression from both self and parents, to clarify familial transmission from different informant sources.

## METHODS

### Participants

Same‐sex twin families with twins in adolescence (*N* = 987; mean age = 13.68 years, *SD* = 2.23 years) were recruited from the Beijing Twin Study (BeTwiSt). Details of the BeTwiSt can be found in a prior study (Chen et al., 2013). We combined DNA analysis and a questionnaire to determine twins' zygosity. This combination method has an accuracy of 99% (Chen et al., 2010). In our sample, there were 716 pairs of MZ twins and 271 pairs of DZ twins. All twins were of Han ethnicity, and 51% were girls. No rewards were offered for their participation.

### Procedures

After school, trained research staff gathered all the twins who wanted to participate in this study in their classrooms. Staff described the study's purposes and procedures, and then distributed the questionnaires to all the participants. First, the questionnaires assessing demographic information and depression symptoms were completed independently by the twins. Next, staff collected saliva samples from the twins to extract their DNA (DNA Genotek Inc., Kanata, ON, Canada). Finally, adolescents were asked to take the questionnaires for parents back home. Parents completed the questionnaires at home and submitted them through their children. Informed consent was obtained from both twins and their parents before their participation. The Scientific Research Ethics Committee at the Institute of Psychology, Chinese Academy of Sciences approved all the procedures.

### Measures

#### Adolescent depression symptoms

In self‐report, twins' depression symptoms were measured using the Children's Depression Inventory (CDI; Kovacs et al., 2003). This 20‐item scale assessed twins' behaviors in the previous 2 weeks, ranging from 1 (*absence of symptoms*) to 3 (*definite symptoms*). Higher scores indicate an increased severity of depression. The CDI has been used previously to measure depression in Chinese adolescents (Chen et al., 2014). In our sample, Cronbach's *α* is .86.

In parent‐report, the CDI Parent Form was used to assess adolescent depressive symptoms (CDI‐PF; Kovacs et al., 2003). CDI‐PF comprises the same items as the original CDI but with reworded versions designed for parents to rate their children. For instance, the item “I feel sad all the time” is rephrased as “My child feels sad all the time.” In this study, Cronbach's *α* was .87. Parent‐reported CDI‐PF data were available for 97.97% of the twins.

#### Parental depression symptoms

All parents completed the self‐report version of the Center for Epidemiologic Studies Depression Scale (CESD; Radloff, 1977). Parents were asked to report their depressive feelings during the last week. This scale comprises 20 items scored from 0 (*less than 1 day*) to 3 (*5 to 7 days*). Higher scores indicate increased frequency. The CESD questionnaire has been used extensively for Chinese adults (He et al., 2019; Xu et al., 2021). Cronbach's *α* in the current study was .85 for mothers and .83 for fathers. Consistent with the NTF model assumptions, we utilized missing values to substitute for parental depression data for parents who did not share 50% of their genes with the twins. Consequently, we had access to biological maternal self‐reported data for 96.45% and to biological self‐reported paternal data for 84.09% of twin families.

#### Covariates

The twins reported their age and sex. Fathers and mothers reported their own age. Sex and age can influence the covariance between family members, potentially introducing bias into the estimates of genetic and environmental influences. As a result, the effects of these two factors were regressed prior to the NTF model analyses (McGue & Bouchard, 1984).

### NTF model analyses

**FIGURE 1:**
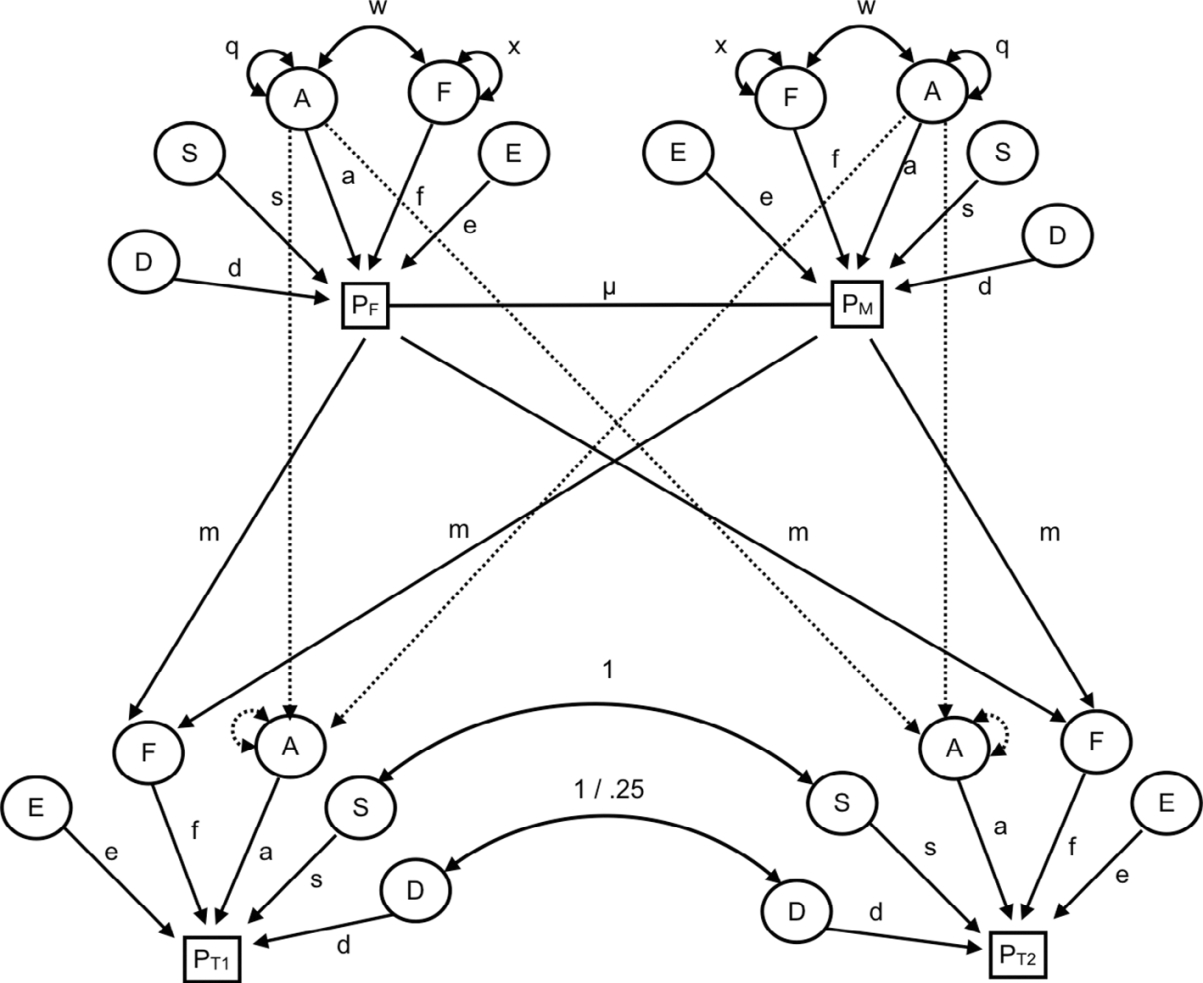
Path diagram of the nuclear twin family model. P_F_, P_M_, P_T1_, and P_T2_ indicate the phenotype variance for fathers, mothers, first‐born twins, and second‐born twins, respectively. A = additive genetic variance; D = dominant genetic variance; S = sibling environmental variance; F = familial environmental variance; E = unique environmental variance; a = additive genetic effects; d = dominant genetic effects; s = sibling environmental effects; f = familial environmental effects; e = unique environmental effects that include measurement errors; m = familial environmental transmission from parents to offspring; μ = assortative mating between twins’ parents; w = covariance between A and F; x = expected variance of latent variable F; q = variance of latent variable A.

The NTF model has demonstrated its utility in elucidating the etiology of a variety of social and psychological factors (e.g., Bleidorn et al., 2018; Burt & Klump, 2012; Ding et al., 2023; Hufer et al., 2020; Slawinski et al., 2019). Figure 1 shows the full NTF model for both MZ and DZ twin families. For genetic influences, the NTF model is able to estimate both A and D. The A between MZ twins is assumed to be perfectly correlated (*r* = 1.00), whereas between DZ twins as well as between parents and offspring, it is assumed to be half‐correlated (*r* = 0.5). The D between MZ twins is perfectly correlated (*r* = 1.00), whereas between DZ twins, it is partially correlated (*r* = 0.25). In terms of environmental influences, the NTF model can estimate the familial environmental influences shared by parents and all children (F), the sibling environmental influences shared only by children (S), and the unique environmental influences (E) that vary among family members. The S between twins is perfectly correlated (*r* = 1). Finally, it can estimate assortative mating (μ) and passive gene–environment correlation (w) simultaneously.

By integrating the depression data of both twins and their parents, the NTF model can gather four kinds of information: the covariance between parents, between parents and their children, between MZ twins, and between DZ twins. The covariance decomposition equations for variances/covariances of interest are shown in Table S1 (Supplementary Material; Keller et al., 2010). The estimates obtained from the NTF model were calculated using the structural equation model, fitted by OpenMx in R, version 4.0.1 (R Foundation for Statistical Computing, Vienna, Austria) (Neale et al., 2016). The NTF model assumes that both A and E influence all phenotypes and includes them in each model. However, owing to the lack of enough information to estimate all these parameters together, D, S, or F must be fixed to zero in a given model. The analytical scripts for the NTF model can be found in the Supplementary Material.

We utilized the standardized residuals of depression data, obtained after regressing out the effects of age and sex, for the analysis. First, we freely estimated the variances, covariances, and means to obtain a baseline index of fit. These calculations were followed by estimating three alternative baseline NTF models (i.e., ASFE, ADFE, and ADSE) and four nested models (i.e., AFE, ASE, ADE, and AE). Specifically, when estimating the AFSE model, dominant genetic effects (d) were set to zero. When estimating the ADFE model, sibling environmental effects (s) were fixed to zero. When estimating the ADSE model, familial environmental transmission (m), passive gene–environment correlation (w), and the expected variance of latent variable F (x) were all fixed to zero. This simplification was applied to create a more concise ADSE model, because w and x could act only if both A and F were involved (Keller et al., 2010). Then, nested models were estimated according to the above rule. Finally, the relative proportions for various genetic and environmental influences were estimated for the optional model. We utilized adolescent depression data reported by the twins themselves and by their parents, respectively, to conduct a NTF model analysis along with parental depression data. We used full‐information maximum‐likelihood raw data techniques to address missing data (Little & Rubin, 2019).

## RESULTS

### Descriptive statistics and Pearson correlations

Table 1 shows the descriptive statistics and correlations between twins' and parental depression. Pearson correlations of depression between family members are all significant, revealing substantial family clustering. Furthermore, correlation coefficients within twin pairs were calculated separately for MZ and DZ twins. In both self‐ and parent‐reported data, the correlations in MZ twins were higher than those in DZ twins, but they were not quite double the correlations observed in DZ twins, indicating genetic, and shared and unique environmental influences on adolescent depression.

**TABLE 1:** Descriptive statistics and Pearson correlations.

	Depression	Mean (*SD*)	Correlation							
			1	2	3	4	5	6	MZ	DZ
1	Twin 1 (Self‐report)	1.37 (0.25)	—						0.51***	0.32***
2	Twin 2 (Self‐report)	1.37 (0.26)	0.45***	—						
3	Twin 1 (Parent‐report)	1.28 (0.20)	0.50***	0.28***	—				0.63***	0.41***
4	Twin 2 (Parent‐report)	1.26 (0.21)	0.45***	0.43***	0.56***	—				
5	Father	0.49 (0.36)	0.10**	0.15***	0.20***	0.26***	—			
6	Mother	0.55 (0.41)	0.13***	0.15***	0.24***	0.26***	0.34***	—		

*Note*: MZ = monozygotic twins; DZ = dizygotic twins; Twin 1 = first‐born twin; Twin 2 = second‐born twin. Self‐report: Twins' depression was reported by the twins themselves; Parent‐report: Twins' depression was reported by parents.

*** *p* < .001.

** *p* < .01.

### NTF model

The model‐fitting results for all NTF models can be found in Table 2. Most NTF models provided at least an acceptable model fit, with the root mean square error approximation (RMSEA) ≤ .06 and the comparative fit index (CFI) ≥ .90. Among these acceptable models, the one with the lowest Akaike information criterion (AIC; Akaike, 1987) was considered the best. In self‐ and parent‐report, the ADSE (m = x = w = 0) model had the lowest AIC, and was thus considered as the optimal model. It indicates that additive and dominant genetic, and sibling and unique environmental influences significantly contributed to adolescent depression. However, familial environmental influences and passive gene–environment correlation did not.

**TABLE 2:** Fit indices of NTF models for adolescent depression via self‐ and parent‐report.

Informant for twins' depression	Model	−2logL	*df*	RMSEA	CFI	AIC
Self‐report	Saturated model	10066.29	3663	0	1	2740.29
	ASFE model (d = 0)	10128.93	3682	0.05	0.88	2764.93
	ADFE model (s = 0)	10117.85	3682	0.04	0.91	2753.86
	**ADSE model (m, w, x = 0)**	**10114.71**	**3684**	**0.04**	**0.92**	**2746.71**
	AFE model (d, s = 0)	10194.91	3683	0.07	0.71	2828.91
	ASE model (m, w, x, d = 0)	10124.68	3685	0.07	0.72	2754.68
	ADE model (m, w, x, s = 0)	10117.86	3685	0.04	0.92	2747.86
	AE model (m, w, x, d, s = 0)	10194.89	3686	0.04	0.9	2822.89
Parent‐report	Saturated model	9611.98	3523	0	1	2365.98
	ASFE model (d = 0)	9668.46	3642	0.04	0.94	2384.46
	ADFE model (s = 0)	9661.73	3642	0.04	0.95	2377.73
	**ADSE model (m, w, x = 0)**	**9661.33**	**3644**	**0.04**	**0.95**	**2373.33**
	AFE model (d, s = 0)	9765.89	3643	0.08	0.77	2479.89
	ASE model (m, w, x, d = 0)	9688.46	3645	0.04	0.94	2378.46
	ADE model (m, w, x, s = 0)	9666.29	3645	0.04	0.95	2376.28
	AE model (m, w, x, d, s = 0)	9750.58	3646	0.07	0.8	2458.58

*Note*: The best‐fitting model via each informant in each wave (as indicated by the lowest AIC) is highlighted in bold. Additive genetic, dominant genetic, sibling environmental, familial environmental, and unique environmental influences are denoted as A, D, S, F, and E, respectively. Self‐report: Depression data for twins were reported by the adolescents themselves, whereas parental depression data were reported by the parents themselves. Parent‐report: Both twins' and parental depression were reported by the parents. d = 0: no dominant genetic effects; s = 0: no sibling environmental effects; m = 0: no environmental transmission from parents to offspring; w = 0: no covariance between A and F; x = 0: no expected variance of the latent variable F.

Abbreviations: AIC = Akaike's information criterion; CFI = comparative fit index; *df* = degrees of freedom; −2logL = −2 log‐likelihood; RMSEA = root mean square error approximation.

The parameter estimates for the best‐fitting NTF model, ADSE (m = x = w = 0), were calculated to elucidate the relative proportions of genetic and environmental contributions (see Table 3). In both self‐ and parent‐report, high genetic influences, including additive and dominant genetic influences, contributed to adolescent depression. Furthermore, a mild but significant sibling environmental influence within twins was found. Unique environmental influences, explaining the individual differences among family members, consistently occupied the largest environmental proportion. The significant assortative mating supports the existence of couple similarity in depression and confirms the importance of including it in the twin model. Despite the relatively consistent results obtained from both sets of informants, some slight differences emerged. Specifically, in the parent‐reported data, total genetic influences were higher, while unique environmental influences were lower compared with those in the self‐reported data.

**TABLE 3:** Parameter estimates for ADSE (m, w, x = 0) model.

Informant for twins' depression	A	D	S	E	Assortative mating
Self‐report	0.21 [0.14, 0.27]	0.22 [0.01, 0.37]	0.12 [0.00, 0.24]	0.46 [0.41, 0.51]	0.35 [0.28, 0.40]
Parent‐report	0.37 [0.30, 0.43]	0.17 [0.04, 0.30]	0.13 [0.02, 0.24]	0.33 [0.30, 0.37]	0.35 [0.29, 0.41]

*Note*: Additive genetic, dominant genetic, sibling environmental, and unique environmental influences are denoted as A, D, S, and E, respectively, and they are all standardized variance components. 95% confidence intervals are presented in square brackets. Self‐report: Depression data for twins were reported by the adolescents themselves, whereas parental depression data were reported by the parents. Parent‐report: Both twins' and parental depression were reported by the parents.

## DISCUSSION

This is the first study to involve data from both adolescent twins and their biological parents to give a comprehensive view on how depression transmits within family members. The findings revealed the significant contributions of additive and dominant genetic influences and sibling environmental influences on adolescent depression. However, no evidence was found for familial environmental influences or passive gene–environmental correlation.

According to both self‐ and parent‐report, approximately 50% of the variance in adolescent depression was attributed to genetic influences, supporting the significant role of genetic transmission. Besides the commonly found additive genetic effects in CT studies (Chen et al., 2014; Eley & Stevenson, 1999; Rice et al., 2002; Scourfield et al., 2003; Zheng et al., 2016), significant dominant genetic effects were also detected. This suggests that the genetic mechanism underlying adolescent depression might be more complex than previously anticipated, with important implications for gene location studies regarding the consideration of gene variant interactions.

The absence of familial environmental influences in our study indicates that there is no evidence for inter‐generational environmental transmission. This suggests that parent–child transmission of depression is primarily due to inherited genetic factors, rather than to shared familial environmental factors. The absence of a passive gene–environmental correlation in our study may contribute to the lack of parent–child environmental transmission. Specifically, despite transmitting risk genes to their children, parents carrying depression‐related genes do not typically create the negative familial environments that influence depression. It is also possible that adolescents are in a vital process of establishing symbolic parental separation. This will lead to a gradual decline in parent–child interactions that can contribute to depression transmission. However, this finding seems to conflict with studies that have found that many kinds of familial environment (e.g., family socioeconomic status and family relationships) influence adolescent depression (Pino et al., 2018; Queen et al., 2013). This discrepancy may be due to the fact that adolescents often perceive these familial environments differently from their parents or siblings (Plomin et al., 2001). Consequently, these factors will not be recognized as familial environmental factors shared by both parents and children, but rather as sibling or unique environmental factors in the twin model. Previous extended twin family research supports this view by revealing that the environmental factors impacting adolescent depression are distinct from those influencing maternal depression symptoms (Rice et al., 2005).

Conversely, our study found significant environmental influences that are shared by siblings. This suggests that the environmental transmission of depression is primarily constrained to the same generation. During adolescence, frequent interactions between siblings occur, and this could lead to a contagion of depressed feelings. For instance, one sibling's discussion about negative feeling and problems can increase the risk of depressive symptoms in the other (Buist et al., 2019). Furthermore, twins often share common environments, including peers, school, and neighborhoods, which can expose them to certain risk factors (e.g., dysfunctional school climate, peer victimization, and neighborhood violence) for depression (Arora et al., 2020; Jia et al., 2009; Mendelson et al., 2010; Yang et al., 2020). The significant within‐generational environmental transmission observed in our study emphasizes the importance of targeting sibling‐shared environments in prevention and intervention programs.

Finally, we found minor differences in genetic and unique environmental influences between self‐ and parent‐report. Depression within parental perceptions was found to be more heritable and less environmental in our study, consistent with previous CT research (Chen et al., 2014; Rice et al., 2002; Scourfield et al., 2003). This discrepancy may be attributed to the fact that parental perceptions primarily occur within the home context, where unique environmental factors outside the home are generally lacking (Bartels et al., 2007). Additionally, parents' own emotional states can impact their reporting of their children's emotions (Lagattuta et al., 2012). When parents experience depression themselves, they are more likely to report more depressive symptoms in their children, potentially leading to an overestimation of genetic influences. However, it is necessary to note that despite these differences, unique environmental influences found in both self‐ and parent‐report are strong. This highlights the significance of parents and clinicians paying more attention to risk environments outside the family.

The strengths of this study include its elaborated behavioral genetic approach, a large representative twin family sample, and the use of multiple informants. Nevertheless, we need to acknowledge certain limitations in our study. First, our results are specific to adolescents. While adolescence is a critical period for the occurrence of depression symptoms, future research is needed to investigate different life periods, such as childhood and adulthood, to extend these findings. Furthermore, the study did not consider complex processes such as the gene–environment interaction (i.e., the dependence of genetic effects on the environment) and evoked gene–environment correlation (i.e., adolescent's depression might influence parents; McAdams et al., 2015). In future studies, it may be beneficial to incorporate additional data sources, such as depression data from grandparents, to address these concerns.

In conclusion, this multi‐informant twin family study has provided insights into how depression transmits within family members. Our results suggest that the familial transmission of depression can be attributed to the genetic influences passed from parents to children and the environmental influences transmitted within siblings. These findings deepen the understanding of the etiology of depression and have important implications for preventing familial transmission in adolescence.

## CONFLICT OF INTEREST STATEMENT

No potential conflict of interest was reported by the authors.

## ETHICS STATEMENT

The Scientific Research Ethics Committee at the Institute of Psychology, Chinese Academy of Sciences, has approved all the procedures (Reference number: H20037).

## Supporting information


**DATA S1:** Supporting Information.
