# Supplementary material for: Familial genetic and environmental transmission of depression: A multi‐informant twin family study
Source: Psych J. 2024 Apr 15;13(5):796–803. doi: 10.1002/pchj.751 (PMC11444726; doi:10.1002/pchj.751)
Supplement: Supplementary file 1 — DATA S1: Supporting Information. [file PCHJ-13-796-s001.docx]

Supplement material

Table S1 *Covariance decomposition for variances/covariances of interest.*

| *V_P_*=*σ^2^*=*a*^2^*q*+*f*^2^*x*+2*awf*+*e*^2^ +*d*^2^ +*s*^2^ |
| --- |
| *V_A_*=*a^2^q, V_D_*=*d*^2^, *V_S_*=*s^2^*, *V_F_*=*f^2^x*, *V_E_*=*e^2^* |
| *CV*(*MZ,MZ*)=*a*^2^*q*+*d*^2^+*s*^2^+*f*^2^*x*+2*awf* |
| *CV*(*DZ,DZ*)=*a*^2^(*q*–1/2)+1⁄4*d*^2^+*s*^2^+*f*^2^*x*+2*awf* |
| *CV*(*Par,Child*)=1⁄2*a*(*qa*+*wf*)+1⁄2*a*(*qa*+*wf*)*μσ^2^*+*m*σ^2^+*mσ^2^μσ^2^* |

require(OpenMx); require(foreign); require(MASS);require(psych)

# Load Data

data <- read.spss("")

# Pick the variables

selVars = c("ZRE_t1selfCDI","ZRE_t2selfCDI","ZRE_faCDI","ZRE_moCDI")

mz.ntf <- subset(data, zyg2012=="MZ", selVars)

dz.ntf <- subset(data, zyg2012=="DZ", selVars)

nv<-1

a <- mxMatrix(type="Lower",nrow=nv,ncol=nv,free=T,values=.6,label="AddGenPath",name="a")

m <- mxMatrix(type="Lower", nrow=nv, ncol=nv, free=F, values=.0, label="FamilialPath", name="m")

d <- mxMatrix(type="Lower",nrow=nv,ncol=nv,free=T,values=.6,label="DomPath",name="d")

s <- mxMatrix(type="Lower", nrow=nv, ncol=nv, free=T,values=.6,label="SibPath", name="s")

e <- mxMatrix(type="Lower",nrow=nv,ncol=nv,free=T,values=.6,label="EnvPath",name="e")

mu <- mxMatrix(type="Lower",nrow=nv,ncol=nv,free=T,values=.3,label="AMCopath",name="mu")

# Matrices for latent variances

Vp1 <- mxMatrix(type="Full",nrow=nv,ncol=nv,free=T,values=1.5,label="VarPhen",name="Vp1")

delta1 <- mxMatrix(type="Full",nrow=nv,ncol=nv,free=T,values=.5,label="CovPhenGen",name="delta1")

q1 <- mxMatrix(type="Full",nrow=nv,ncol=nv,free=T,values=1.2,label="LatentVarAddGen",name="q1")

w1 <- mxMatrix(type="Full", nrow=1, ncol=1, free=TRUE, values=0, label="CovFA",name="w1")

x1 <- mxMatrix(type="Full", nrow=1, ncol=1, free=TRUE, values=0, label="VarF",name="x1")

# mxAlgebra - nonlinear constraints

Vp2 <- mxAlgebra(a %&% q1 + x1 + 2 %x% a %*% w1 + e %*% t(e) + d %*% t(d) + s %*% t(s),name="Vp2")

delta2 <- mxAlgebra(q1 %*% a + w1,name="delta2")

q2 <- mxAlgebra(1 + delta1 %&% mu,name="q2")

x2 <- mxAlgebra(2 %x% m %&% Vp1 + 2 %x% m %*% m %*% Vp1 %*% Vp1 %*% mu, name="x2")

w2 <- mxAlgebra(delta1 %*% m + delta1 %*% mu %*% Vp1 %*% t(m), name="w2")

#variance components

VA <- mxAlgebra(expression=a %&% q1, name="VA")

VD <- mxAlgebra(d %*% t(d), name="VD")

VE <- mxAlgebra(e %*% t(e), name="VE")

VAW <- mxAlgebra(2 %x% a %*% w1,name="VAW")

VS <- mxAlgebra(s %*% t(s), name="VS")

V <- mxAlgebra(VA+VD+VE+VS+VAW,name = "V")

# standardized variance components

a2 <- mxAlgebra(VA/Vp1, name = "a2")

d2 <- mxAlgebra(VD/Vp1, name = "d2")

e2 <- mxAlgebra(VE/Vp1, name = "e2")

s2 <- mxAlgebra(VS/Vp1, name = "s2")

aw <- mxAlgebra(VAW/Vp1, name = "aw")

# Equating nonlinear constraints and parameters

VpCon <- mxConstraint(Vp1==Vp2,name="VpCon")

deltaCon <- mxConstraint(delta1==delta2,name="deltaCon")

qCon <- mxConstraint(q1==q2,name="qCon")

wCon <- mxConstraint(w1==w2,name="wCon")

xCon <- mxConstraint(x1==x2,name="xCon")

# mxAlgebra - relative covariances

CvMz <- mxAlgebra(a %&% q1 + x1 + 2 %x% a %*% w1 + d %*% t(d) + s %*% t(s),name="CvMz")

CvDz <- mxAlgebra(a %&% (q1-.5) + .25 %x% d %*% t(d) + x1 + 2 %x% a %*% w1 + s %*% t(s),name="CvDz")

ParChild <- mxAlgebra(.5 %x% a %*% delta1 + .5 %x% a %*% delta1 %*% mu %*% Vp1 + m %*% Vp1 + m %*% Vp1 %&% mu, name="ParChild")

CvSps <- mxAlgebra(Vp1 %&% mu,name="CvSps")

#Put these relative covariances together into MZ relatives and DZ relatives matrices

MZ.rel.cv <- mxAlgebra(rbind(

cbind(Vp1, CvMz, ParChild, ParChild),

cbind(CvMz, Vp1, ParChild, ParChild),

cbind(ParChild, ParChild, Vp1, CvSps),

cbind(ParChild, ParChild, CvSps, Vp1)),

dimnames=list(selVars,selVars),name="expCovMZRels")

DZ.rel.cv <- mxAlgebra(rbind(

cbind(Vp1, CvDz, ParChild, ParChild),

cbind(CvDz, Vp1, ParChild, ParChild),

cbind(ParChild, ParChild, Vp1, CvSps),

cbind(ParChild, ParChild, CvSps, Vp1)),

dimnames=list(selVars,selVars),name="expCovDZRels")

#Put the relatives means together into means matrix (same for MZ and DZ rels)

Both.means <- mxMatrix(type="Full", nrow=1, ncol=4, free=TRUE, values=.05,label= paste("mean",1:4,sep=""),name="mean")

expMeanBoth <- mxAlgebra(mean, name="expMeanBoth")

# Put in the data

dataMZ <- mxData( observed=mz.ntf, type="raw" )

dataDZ <- mxData( observed=dz.ntf, type="raw" )

#Objective objects for two groups

objMZRels <- mxExpectationNormal(covariance="expCovMZRels",means="expMeanBoth", dimnames=selVars)

objDZRels <- mxExpectationNormal(covariance="expCovDZRels",means="expMeanBoth", dimnames=selVars)

#Combine groups

myfitfun <- mxFitFunctionML()

params <- list(a,d,s,e,mu,m,Vp1,delta1,q1,x1,w1,Vp2,delta2,q2,x2,w2,VpCon,deltaCon,qCon,xCon,wCon,CvMz,CvDz,ParChild,CvSps,MZ.rel.cv,Both.means,expMeanBoth,DZ.rel.cv,myfitfun,VA,VD,VE,VAW,V,VS,a2,d2,s2,aw,e2)

params1 <- list(a,d,s,e,mu,m,Vp1,delta1,q1,x1,w1,Vp2,delta2,q2,x2,w2,CvMz,CvDz,ParChild,CvSps,MZ.rel.cv,Both.means,expMeanBoth,DZ.rel.cv,myfitfun,VA,VD,VE,VAW,V,VS,a2,d2,s2,aw,e2)

ci2 <- mxCI(c('VA','VD','VE','VAW','VS','a2','d2','s2','aw','e2'))

modelMZ <- mxModel("MZntf",params1,dataMZ,objMZRels,ci2)

modelDZ <- mxModel("DZntf",params,dataDZ,objDZRels)

obj <- mxFitFunctionMultigroup(c("MZntf","DZntf"))

ci1 <- mxCI(c("AddGenPath","SibPath","DomPath","EnvPath","AMCopath","FamilialPath","VarPhen","CovPhenGen","LatentVarAddGen", "VarF","CovFA","SibPath"))

ADSE.NTF.Model <- mxModel("ntfADSE",modelMZ,modelDZ,obj,ci1)
